# Influence of perceived parental child-rearing attitudes and ego identity on college adjustment among Korean nursing students

**DOI:** 10.1186/s12912-023-01643-9

**Published:** 2024-01-02

**Authors:** Hye Seon Choi, Sona Lee, Eunju Seo

**Affiliations:** 1 College of Nursing, Woosuk University, Wanju, 55338 Republic of Korea; 2 College of Nursing, Eulji University, Uijeongbu, 11759 Republic of Korea; 3 Department of Nursing, Pai-Chai University, Daejeon, 35345 Republic of Korea

**Keywords:** Adaptation, Ego identity, Parental Child-rearing Attitude, Parent–Child Relations, Students, Nursing

## Abstract

**Background:**

This study aimed to examine the relationship between nursing students’ perceived parental child-rearing attitude, ego identity, and college adjustment in Korea and explore factors that influence college adjustment.

**Methods:**

This study surveyed 224 nursing students enrolled in universities located in two regions within South Korea. Data were collected from October 14 to November 31, 2019. Perceived parental child-rearing attitude (paternal emotional warmth, paternal rejection, paternal overprotection, maternal emotional warmth, maternal rejection, and maternal overprotection) and ego identity of nursing students were used as independent variables on college adjustment. Collected data were subjected to correlation analysis using SPSS version 26.0 for Windows. Further, regression analysis was performed on the influence of parental child-rearing attitude and ego identity on college adjustment.

**Results:**

Among the parental child-rearing attitudes, paternal emotional warmth (r = .30, *p* < .001), maternal emotional warmth (r = .38, *p* < .001), and ego identity (r = .71, *p* < .001) were positively correlated with nursing students’ college adjustment, whereas maternal rejection was negatively correlated with ego identity (*r* = − .28, *p* < .001) and college adjustment (r = − .15, *p* = .025). Regression analysis of the effects of nursing students’ perceived parental child-rearing attitude and ego identity on college adjustment, with grade as a control variable, revealed that ego identity (*p* < .001) had a significant effect on college adjustment, and the higher the ego identity (β = 0.712), the higher the college adjustment. Further, the explanatory power of explaining college adjustment was high at 49.9%.

**Conclusions:**

The nursing students’ perceived paternal emotional warmth, maternal emotional warmth, and ego identity were positively correlated with college adjustment. Additionally, ego identity was found as the influencing factor in Korean nursing students’ college adjustment. Therefore, programs to strengthen ego identity should be developed and implemented for college adjustment among nursing students.

## Background

Nurses work in various settings, including hospitals, clinics, schools, and home health agencies. Moreover, they work closely with other health care professionals, including doctors, pharmacists, and social workers, to provide comprehensive health care services to patients [1], as well as education and emotional support to patients and their families. As such, nurses play a unique and essential role in health care [1, 2].

In South Korea, the number of nurses working in health care organizations increased from 2.4 to 4.4 per 1,000 people over the past decade (from 2011 to 2020) [3, 4]. The number of nursing school enrollments in South Korea has also increased over the past decade. In 2020, the number of students enrolled in nursing education institutions per 1,000 population was 0.4 and continues to increase [5].

While the number of students enrolled in nursing programs has increased, the number of those who drop out, either by taking a leave of absence or dropping out, has also escalated [6, 7]. Nursing students, like other college students, may experience maladjustment during the transition from high school life to college life, which is centered on admissions. Common causes of maladjustment experienced by nursing students include academic stress, homesickness, financial difficulties, lack of social support, parental child-rearing attitude, and difficulty adapting to a new environment [8, 9]. In particular, nursing students experience anxiety and increased stress during their university life due to the heavy academic workload, more than 1,000 h of clinical practice, and preparation for the national nursing examination to acquire specialized knowledge in nursing.

Maladjustment can affect their physical and mental health, academic achievement, and overall college life of nursing students and must be prevented and managed. Moreover, maladjustment in nursing students increases stress, anxiety, and depression [10], which can negatively impact their academic success and future careers [11]. Furthermore, maladjustment in nursing students can have broader implications for the health care industry as a whole [9] considering that nursing students who struggle to adjust to college are more likely to drop out or perform poorly [12], which can lead to a societal problem in the form of a future shortage of qualified health care workers [4]. Therefore, addressing the problem of maladjustment in nursing students is of considerable importance.

Research on the adjustment of college nursing students needs to examine various factors. In particular, internal and external factors need to be explored in such studies.

Parenting attitudes (an individual's extrinsic factors) refer to how an individual perceives parenting practices, such as discipline, communication, and warmth [13]. Another paper defines parenting attitudes as an individual's subjective perception of how their parents treated them and how their parenting style influenced their personality traits, beliefs, values, and behaviors [14]. Nursing students’ perceived parenting attitudes may influence their beliefs, values, and behaviors as health care professionals [3]. Ego identity (an individual's intrinsic factors) is the perception of oneself as a unique and distinctive being, reflecting on not only one's own views of one's own characteristics and values but also the views of others [15]. Ego identity begins to form and develop in childhood and is fulfilled during the psychosocial crises experienced during the college years as one transitions from adolescence to adulthood [16]. Nurse identity begins to form during the college years after entering nursing school and continues to develop throughout the nursing profession [17]. As such, parenting attitudes and ego identity are important intrinsic and extrinsic factors in understanding nursing students' adjustment to college [18, 19].

Therefore, this study aimed to examine the association of parental child-rearing attitudes (external factors) and ego identity (internal factors) with college adjustment among nursing students and explore the factors that influence nursing students’ college adjustment in Korea. Furthermore, based on the results of this study, we hope to provide a better understanding of nursing students' college adjustment and basic data for the development of programs to improve nursing students' college adjustment.

## Methods

### Study design

This exploratory investigation study determined the influence of Korean nursing students’ perceived parental child-rearing attitude and ego identity on college adjustment.

### Data collection and ethics

Before data collection, the survey was approved (IRB#: EU19–82) by the Institutional Review Board (IRB) of Eulji University to which the researcher belongs. Data were collected from 14 October to 31 November 2019. This study was conducted among nursing students enrolled in universities located in cities D and J, South Korea. The researcher visited each university to explain the study’s purpose, need, and data collection procedure; to obtain cooperation from university officials; and to survey those who agreed to participate in the study. The questionnaires were handed out in person, and the self-completed questionnaires were collected by the researcher in person. The sample size was calculated using G*Power version 3.1.9.2, a sample size calculation software. Considering an effect size of 0.4, significance level of 0.05, and power of 0.80 required for correlation analysis [20], a minimum of 204 subjects were needed. Considering the 15% dropout rate, 235 people were surveyed with a return rate of 95%, and 224 copies were used for the final analysis.

### Measurements

#### Parental child-rearing attitude

Parental child-rearing attitude was measured using the s-EMBU instrument [21] developed by Arrindell and adapted into a Korean version by Jo [22]. This instrument consists of three subcategories (parental emotional warmth, parental rejection, and parental overprotection). This instrument consists of 23 items and is scored using a 4-point Likert scale. Each item is measured on a scale from 1, “No, never,” to 4, “Yes, most of the time,” with higher scores indicating higher characteristics of that subdomain. In the Arrindell study, the Cronbach's alpha of the instrument was 0.79 for paternal emotional warmth, 0.79 for maternal emotional warmth, 0.76 for paternal rejection, 0.77 for maternal rejection, 0.80 for paternal over protection, and 0.82 for maternal over protection [21]. In Jo's study, the Cronbach's alphas of the instruments were 0.88 for parental emotional warmth, 0.75 for parental rejection, and 0.72 for parental overprotection [22]. In the current study, the Cronbach's alpha of the parental child-rearing instrument was 0.82 for paternal emotional warmth, 0.80 for maternal emotional warmth, 0.81 for paternal rejection, 0.82 for maternal rejection, 0.078 for paternal over protection, and 0.79 for maternal over protection.

### Ego identity

Ego identity was measured using the Korean version of the ego identity scale developed by Park [23] and revised by Park [24]. This instrument consists of six subcategories (initiativeness, self-receptiveness, confirmativeness for future, goal orientedness, subjectivity, and intimacy). The tool consists of 60 items and is scored on a 5-point Likert scale. Each item is measured on a scale from 1 (Not applicable at all) to 5 (It applies very much), with higher scores indicating higher ego identity. The Cronbach's alpha of the revised ego identity instrument was 0.94 [24]. In the current study, the Cronbach's alpha of the ego identity instrument was 0.96.

### College adjustment

College adjustment was measured using the college adjustment scale developed by Jeong and Park [25]. This instrument consists of five subcategories (interpersonal relations, academic activities, career preparation, personal psychology, and social participation). The instrument consists of 19 items and is scored on a 5-point Likert scale. Each item is measured from 1 (not at all) to 5 (very much so), with higher scores indicating higher adjustment to college. The Cronbach's alpha of the instrument was 0.86 in Jeong & Park's study and 0.90 in this study [25].

### Data analysis

The collected data were analyzed using SPSS (IBM Corporation, New York, USA) for Windows. The reliability of the scales was verified using Cronbach’s α. General characteristics are presented using frequencies and percentages, whereas parental child-rearing attitude, ego identity, and college adjustment are presented using descriptive statistics (mean and standard deviation). College adjustment according to the general characteristics of nursing students was analyzed using the t-test and analysis of variance (ANOVA), and a post-hoc analysis was conducted using Scheffé's test. Correlations between parental child-rearing attitude, ego identity, and college adjustment were analyzed by Pearson's correlation coefficient. A controlled regression analysis was conducted on the effects of grade, as a control variable, and parental child-rearing attitude and ego identity, as independent variables, on college adjustment of nursing students.

## Results

### Participant demographics and characteristics

The general characteristics of the subjects included in this study are summarized in Table 1. There were 193 (86.2%) and 31 (13.8%) female and male students, respectively. According to grade level, 61 (27.2%), 56 (25.0%), 55 (24.6%), and 52 (23.2%) were juniors, sophomores, seniors, and freshmen. The most common religious affiliation was none at 138 (61.7%). The education levels of the students’ parents were as follows: college graduate, high school graduate or less, and graduate school or more (Table 1).

**Table 1:** Participants’ characteristics

		(*N* = 224)
**Variables**	**Categories**	**n (%)**
Sex	Female	193(86.2)
	Male	31(13.8)
Grade	1st	52(23.2)
	2nd	56(25.0)
	3rd	61(27.2)
	4th	55(24.6)
Religion	Christian	46(20.5)
	Catholic	24(10.7)
	Buddhist	16(7.1)
	None	138(61.7)
Father′s education	≤ High school	66(29.5)
	College/University	130(58.0)
	> College/University	28(12.5)
Mother′s education	≤ High school	86(38.4)
	College/University	124(55.4)
	> College/University	14(6.2)

### Differences in college adjustment according to participant characteristics

The variable with a significant difference in college adjustment according to the general characteristics of the participants was grade (F = 6.79, *p* < 0.001; Table 2).

**Table 2:** Differences in college adjustment according to participant characteristics

					(*N* = 224)
**Variables**	**Categories**	**Mean**	**SD**	**t or F**	***p***
Sex	Female	66.02	12.17	− 0.50	.647
	Male	67.06	9.31		
Grade	1st ^a^	66.37	10.82	6.79	< .001
	2nd ^b^	60.50	13.45		b < c,d ^*^
	3rd ^c^	68.38	10.64		
	4th ^d^	69.27	10.28		
Religion	Christian	68.91	9.62	1.56	.187
	Catholic	66.00	13.17		
	Buddhist	70.13	13.27		
	None	64.79	11.98		
Father′s education	≤ High school	66.91	10.43	0.43	.730
	College/University	65.51	12.50		
	> College/University	67.18	11.98		
Mother′s education	≤ High school	66.10	11.05	0.51	.602
	College/University	65.85	12.23		
	> College/University	69.21	12.77		

^*^p < .050 by Scheffé’s test

In this study, a significant difference in college adjustment was observed according to grade level (F = 6.79, *p* < 0.001). Post-hoc tests showed that third- and fourth-year students had significantly better college adjustment than did second-year students (Table 2).

### Correlation among research variables

Correlations between parental child-rearing attitude (paternal emotional warmth, paternal rejection, paternal over protection, maternal emotional warmth, and maternal rejection, maternal over protection), ego identity, and college adjustment are shown in Table 3.

**Table 3:** Correlations among the measured variables

								(*N* = 224)	
**Variables**		**Paternal child-rearing attitude**			**Maternal child-rearing attitude**			**Ego identity r(*p*)**	**College Adjus ment r(*p*)**
		**Emotional warmth r(*p*)**	**Rejectionr(*p*)**	**Over protection r(*p*)**	**Emotionalwarmth r(*p*)**	**Rejection r(*p*)**	**Over protection r(*p*)**		
F^a^	**Emotional warmth**	1							
	**Rejection**	− .29 (< .001)	1						
	**Over protection**	.19 (.005)	.51 (< .001)	1					
M^b^	**Emotional warmth**	.66 (< .001)	− .27 (< .001)	− .02 (.770)	1				
	**Rejection**	− .22 (.001)	.67 (< .001)	.39 (< .001)	− .47 (< .001)	1			
	**Over protection**	.07 (.269)	.25 (.001)	.64 (< .001)	− .01 (.899)	.40 (< .001)	1		
**Ego identity**		.46 (< .001)	− .24 (< .001)	− .09 (.178)	.56 (< .001)	− .28 (< .001)	− .08 (.246)	1	
**College adjustment**		.30 (< .001)	− .09 (.172)	− .06 (.337)	.38 (< .001)	− .15 (.025)	− .07 (.281)	.71 (< .001)	1

^a^Paternal child-rearing attitude

^b^Maternal child-rearing attitude

College adjustment was significantly positively correlated with paternal emotional warmth (*r* = 0.30, *p* < 0.001), maternal emotional warmth (*r* = 0.38, *p* < 0.001), and ego identity (*r* = 0.71, *p* < 0.001) but significantly negatively correlated with maternal rejection (r = -0.15, *p* = 0.025; Table 3).

### Regression analysis of factors that influence college adjustment

Regression analysis of the effects of parental child-rearing attitude (paternal emotional warmth, paternal rejection, paternal overprotection, maternal emotional warmth, maternal rejection, and maternal overprotection) and ego identity on college adjustment with grade as a control variable revealed the significant effect of ego identity (*p* < 0.001) on college adjustment, and the higher the ego identity (β = 0.712), the higher the college adjustment. Further, the explanatory power of explaining college adjustment was high at 49.9% (Table 4).

**Table 4:** Factors that influence on college adjustment in nursing students

	$\upbeta$	SE	t	*p*
Constant		5.185	1.721	.087
Grade	0.032	0.522	0.665	.507
Paternal emotional warmth	-0.024	0.166	-0.309	.757
Paternal rejection	0.083	0.313	1.016	.311
Paternal over protection	-0.030	0.228	-0.374	.709
Maternal emotional warmth	0.061	0.193	0.780	.436
Maternal rejection	0.048	0.287	0.601	.548
Maternal over protection	-0.035	0.179	-0.507	.612
Ego identity	0.712	0.020	12.222	.000

Adj R^2^ = 0.499, F = 28.790 (*p* < .001), Durbin-Watson = 1.838

*Β* standardized regression coefficient, *SE* Standard error of the regression coefficient

## Discussion

This study examined the relationship between an individual's external (i.e., parental child-rearing attitude) and internal (i.e., ego identity) factors and college adjustment among nursing students and explored the factors that influence college adjustment among nursing students in South Korea.

After examining the association between nursing students’ perceived parental child-rearing attitude and college adjustment, we revealed that paternal and maternal emotional warmth were significantly and positively correlated with college adjustment. Affectionate parental child-rearing attitudes positively affect college adjustment and help college students become more accepting, respectful, and confident in themselves [18]. Further, they help increase the psychological well-being of college students and improve their interpersonal problem-solving skills [26]. In particular, parental emotional warmth (as a subcategory of parental child-rearing attitudes) provides support and stability for college students in coping with stress and emotional problems while promoting their inner maturity and development [27]. Based on these previous studies and the results of this study, we confirm that parental support and affection are positively related to college students’ college adjustment. Conversely, the current study revealed that nursing students’ perceived maternal rejection was negatively correlated with college adjustment. Arrindell’s research presented a theoretical model in which inappropriate parenting behaviors make children anxious in social situations and contribute to social maladjustment [28]. Several studies confirmed this observation, which has revealed that parental rejection, as perceived by children, causes negative outcomes, such as social anxiety, psychological maladjustment, and smartphone addiction [29–31]. We cautiously speculate that perceived parental rejection causes anxiety in nursing students and may influence college maladjustment, which is the social life of college students. College maladjustment increases the likelihood of dropping out of college [32]. This could disrupt plans to secure a nationally important nursing workforce, which has recently become a major issue in Korean society [31]. Therefore, variables associated with parenting attitudes should be considered when building a system to help nursing students adapt to college life at the college education level.

This study found that the external support system of these individuals, especially parental emotional warmth in the closest relationship, enhanced nursing students' ego identity and improved their adjustment to college. Parental support helps college students trust themselves and take responsibility for their actions, which is consistent with previous research showing that parental support and affection enhance ego identity[18, 33]. For example, universities should engage mothers and provide programs, such as education, counseling, and faculty–parent–student meetings that could improve parenting attitudes. Such programs could provide opportunities for sustaining and enhancing positive parenting attitudes throughout the college years, an early period of ego-identification. In addition, these programs can help nursing students adjust to college. Furthermore, nursing students are also potential future parents; thus, including content related to parenting attitudes in the child and maternal nursing curricula of nursing students is desirable in the long run [34].

In this study, nursing students' ego identity was found to be positively related to college adjustment, and it was identified as an influential factor in college adjustment through regression analysis This is consistent with the findings of Kwon [35], who reported that college students' ego identity was positively correlated with college adjustment and a factor in explaining college adjustment. College students with a high ego identity tend to have an excellent perception of their own personality and worth, recognize their own competence, and demonstrate high ego identity [35]. Moreover, such students are able to establish a realistic view of themselves and plan for the future based on this view [36]. This disposition has a positive impact on college adjustment [36]. In addition, college students with a high ego identity may have better social and relationship skills given that they are more active in their relationships with others and can resolve interpersonal problems amicably [37]. In short, college students with high ego identity can have a positive impact on their adjustment to college. Therefore, we recommend that colleges and universities provide programs to increase college students’ ego identity in various ways. In particular, they can actively utilize student counseling centers, learning support centers, and employment support centers, as well as provide programs for stress management and mentoring programs. These supports can ultimately improve the ego identity of college students, which in turn can improve their college adjustment [38, 39].

The study revealed a positive relationship between parenting attitudes and children’s ego identity. Further, ego identity was an influencing factor on nursing students. As adults, college students become independent of their parents and can psychologically separate their values and beliefs from their parents and make independent choices to live their own lives [40]. Although this process may lead to conflicts with parents, it also allows them to form and strengthen their ego identity [35]. During this time, parents need to respect and support their child's choices to help them develop independence and establish their ego identity, recognize their child's ability to be competent and independent, and provide appropriate support when needed rather than be overly interfering. Parents should have an appropriate child-rearing attitude to help their children establish their ego identity. An appropriate parenting attitude is one that is affectionate, supportive, and trusting of the child, not rejecting or overprotective [30]. Parental roles and appropriate parenting attitudes can be achieved through ongoing education.

Several studies focused on parenting attitudes from infancy to adolescence [41]. However, in Korea, no significant study has focused on parenting attitudes or parental education for parents of college students who have become adults. However, parents need to learn attitudes to make their children independent at the time of adulthood. Therefore, parenting attitudes and planning programs that can influence parental attitudes for parents of college students requires attention. This attention and application of the program can directly or indirectly affect the formation of ego identity in college students.

### Limitations

This study was conducted with a sample of nursing students from universities located in two regions of Korea; hence, there may be limitations in the generalization of the results. Nevertheless, the subjects included herein were enrolled in a nursing college (Baccalaureate) accredited by the Korean Nursing Education Accreditation Commission. As such, these findings are reflective of nursing education in the province and appear congruent with global findings. However, as this is an exploratory study, further research needs to be conducted with a larger sample of nursing students. Furthermore, this cross-sectional study, conducted at a single point in time, makes it challenging to determine causal relationships. Researchers should interpret and generalize the results with caution, considering these limitations. We indicate that future studies combining them with longitudinal research or experimental designs can provide more robust insights.

## Conclusions

The current study was conducted to determine the relationship between perceived parental child-rearing attitudes, ego identity, and college adjustment among nursing students and explored the factors that influence nursing students’ adjustment to college in South Korea. Among the perceived parental child-rearing attitudes of Korean nursing students, paternal emotional warmth, maternal emotional warmth, and ego identity were positively correlated with college adjustment. In addition, among the perceived parental child-rearing attitudes of nursing students, maternal rejection was negatively correlated with ego identity and college adjustment. Further, ego identity was found as the influencing factor in nursing students’ college adjustment. The significance of this study lies in its further confirmation of the relationship between ego identity, an internal factor, and parental child-rearing attitude, an external factor, in exploring factors affecting college adjustment of nursing students. The results of this study may be helping develop programs to improve ego identity and appropriate parental child-rearing attitude education for successful college adjustment of nursing students. Additionally, this study provides foundational evidence to improve diversity and global health nursing education through the lenses of prospective healthcare providers.

We hope that the study will be replicated with nursing students in other countries and that further research will be conducted on various factors affecting nursing students' college adjustment. We also suggest that nursing colleges develop a systematic program to positively influence parental child-rearing attitudes and a program to enhance ego identity.

## Data Availability

The data that support the findings of this study are available from Eulji IRB but restrictions apply to the availability of these data, which were used under license for the current study, and so are not publicly available. Data are, however, available from the authors upon reasonable request and with permission of Eulji IRB.
